# Establishment and validation of a predictive model for mortality within 30 days in patients with sepsis-induced blood pressure drop: A retrospective analysis

**DOI:** 10.1371/journal.pone.0252009

**Published:** 2021-05-20

**Authors:** Bin Wang, Jianping Chen

**Affiliations:** Affiliated Dongyang Hospital of Wenzhou Medical University, Dongyang, Jinhua, Zhejiang Province, China; Universidad Miguel Hernandez de Elche, SPAIN

## Abstract

**Objectives:**

To establish and validate an individualized nomogram to predict the probability of death within 30 days in patients with sepsis-induced blood pressure drop would help clinical physicians to pay attention to those with higher risk of death after admission to wards.

**Methods:**

A total of 1023 patients who were admitted to the Dongyang People’s Hospital, China, enrolled in this study. They were divided into model group (717 patients) and validation group (306 patients). The study included 13 variables. The independent risk factors leading to death within 30 days were screened by univariate analyses and multivariate logistic regression analyses and used for Nomogram. The discrimination and correction of the prediction model were assessed by the area under the Receiver Operating Characteristic (ROC) curve and the calibration chart. The clinical effectiveness of the prediction model was assessed by the Decision Curve Analysis (DCA).

**Results:**

Seven variables were independent risk factors, included peritonitis, respiratory failure, cardiac insufficiency, consciousness disturbance, tumor history, albumin level, and creatinine level at the time of admission. The area under the ROC curve of the model group and validation group was 0.834 and 0.836. The P value of the two sets of calibration charts was 0.702 and 0.866. The DCA curves of the model group and validation group were above the two extreme (insignificant) curves.

**Conclusions:**

The model described in this study could effectively predict the death of patients with sepsis-induced blood pressure drop.

## Introduction

Sepsis is a severe and life-threatening condition due to the response of the body to an uncontrolled infection, which affects millions of people worldwide each year [1–6]. In the United States, more than 970,000 patients suffering from sepsis are hospitalized each year, a number that increases every year [7]. A 20-year study on hospitalized patients in the United States revealed that the incidence of sepsis increases by 8.7% each year [8]. In addition, sepsis accounts for more than 50% of hospital deaths [9], demonstrating the impacts of sepsis on human health. Sepsis is associated with a very high mortality rate [8, 10–12]. Two multicenter studies showed that the in-hospital mortality rate of severe sepsis in China were 48.7% and 35.5%, respectively. Of course, these percentages were dependent on the level of medical care in each region [13, 14]. However, the mortality rate of sepsis and septic shock in China decreased thanks to medical advancements. The available current results revealed that the mortality rate of hospitalized patients with septic shock is approximately 40% [15]. Overall mortality of sepsis is approximately 17%, which is also high [3]. Among the patients with sepsis, some patients with decreased blood pressure did not meet the standard of septic shock, and owned a high mortality rate.

Currently, the clinical evaluation of the severity score and mortality estimation in patients with sepsis-induced blood pressure drop is based on the use of similar scoring tools such as Acute Physiology and Chronic Health Evaluation (APACHEII), or the Sequential Organ Failure Assessment (SOFA) [16, 17]. They all had certain deficiencies in the evaluation of patients with sepsis-induced blood pressure drop. The APACHEII score is applicable to critically ill patients and has a specific ability to predict the probability of death, but it is not specific to patients with sepsis-induced blood pressure drop and does not provide a specific risk assessment for patients with sepsis-induced blood pressure drop. In addition, early established APACHEII score would not be suitable for current updated treatment measures in sepsis patients as before [18]. SOFA score is not used to predict the outcome, but rather to describe the development of Multiple Organ Dysfunction Syndrome [17]. Hence, a lack of tools exists in clinical practice to predict the mortality in a short term of patients with sepsis-induced blood pressure drop.

For the above reasons, it is necessary to develop a tool to predict the mortality in the short term of patients with sepsis-induced blood pressure drop. Therefore, the purpose of this study was to establish a reliable and accurate risk prediction model for patients with sepsis-related blood pressure drop to determine the short-term progression of the disease to death. The prediction model was displayed as a nomogram [19], which could not only visually evaluate respective death risk based on individual variables, but also quantified the death risk of involved patients combining all the risk factors. Furthermore, this model might provide a basis for clinicians for an appropriate clinical evaluation of the patient, a better development of the interventional measures to apply and to promote the communication between clinicians and patients’ families.

## Materials and methods

### Patient selection

Inclusion criteria: (1) Patients with sepsis defined according to the definition of sepsis 3.0, such as: organ dysfunction triggered by infection that endangers the patient’s life. Rapid increase in the sequential (sepsis-related) organ failure assessment score (SOFA), with a total score 2 points. (2) The criteria to evaluate blood pressure reduction were a systolic blood pressure drop of 40 mm Hg from the original basal blood pressure or from the normal blood pressure, or a systolic blood pressure of less than 90 mm Hg at the time of admission to wards (excluding the emergency department). Exclusion criteria: (1) Patients with leukemia, liver cirrhosis, aplastic anemia, human immunodeficiency, autoimmune diseases (there were abnormal levels of white blood cell, red blood cell and platelet in patients with mentioned diseases above, resulting into different variables at baseline comparing to involved participants and higher risk of sepsis and septic shock. The patients with above diseases could be defined through examination results and counseling medical history, and then be excluded), primary peritonitis, other causes leaded shocks (hypovolemic hypotension; anaphylactic reaction due to drugs usage in emergency departments; hypotension due to analgesic drugs in emergency departments), pancreatitis or other diseases at admission; (2) Patients under the age of 18 years; (3) Patients with missing data (including two status on medical records we could not obtained the death results: transfer to another hospital and unhealed when discharged).

We started collecting data for the purpose of this study on April 1, 2020. A final number of 1023 of patients with blood pressure drop caused by sepsis and admitted to Dongyang People’s Hospital from January 2010 to February 2020 were enrolled in this study and retrospectively analyzed. The treatment was performed following standard procedures, such as the administration of anti-infective drugs, fluid infusion, nutritional support, drainage and other forms of treatment. The death was determined based on clinical records when patients were discharged from hospital. If patients were dead in hospital, the status at the time of discharging was “death”; if patients left hospital with improvement in clinical manifestations, their status was recorded as survivor in 30 days; if patients were not cured, they would be recorded as unhealed and excluded in this study.

### Ethics statement

This study was approved by the Ethics Committee of Dongyang People’s Hospital, and written informed consent was obtained from the enrolled patients or their relatives. Also, written informed consent was obtained from guardians of the minors included in the study. The data were anonymously analyzed because the personal information was completely removed. This study was conducted in accordance with the principles of the *Helsinki Declaration* and its subsequent amendments.

### Risk factors involvement and establishment of prediction model

We collected and the following factors of the subjects: Gender, age, and the level of the following indicators at admission: high-sensitivity C-reactive protein (mg/L), creatinine level (mmol/L), albumin level (g/L), platelet level (10*9/L), white blood cells number (10*9/L) and bilirubin (μmol/L); Cardiac insufficiency (cardiac function class III or above, considering the New York Heart Association classification as the standard), respiratory failure (oxygen partial pressure/oxygen concentration less than or equal to 300 mm Hg), peritonitis (diagnosed mostly in emergency department before admission into wards) requiring surgery (including intestinal perforation, intestinal obstruction, and appendicitis), malignant tumor history and consciousness disturbance (such as drowsiness, lethargy, coma) following admission in patients with sepsis-related blood pressure drop. Moreover, heart function and consciousness were evaluated at time of admission, when blood examination results were obtained in first test within 24 hours after admission.

All the evaluations were performed using the R software. The included patients were divided into the model group (n = 717) and the validation group (n = 306) by using createDataPartition function in R software [20]. In the model group, thirteen variables were analyzed by univariate analyses using twogrps function. The significant variables in univariate analysis were included in multivariate logistic regression analysis, before that linearity test was performed. A P greater than 0.1 was considered as linear by boxTidwell function. The forward stepwise method was used to select the variables and were eventually included in the model.

Based on the regression coefficients of independent variables, we established the individualized nomogram prediction model of the short-term mortality in patients with sepsis-related blood pressure drop [21, 22]. First, the individual point for involved risk factors could be obtained by matching the results of variables and point line. Then, all the respective points were summed as total point. Finally, results could be readout by matching the total point and the risk rate of death. The model was evaluated according to three parameters: discrimination, correction and clinical effect.

### Evaluation of established prediction model

The discrimination of the predictive model was defined as the ability of the model to distinguish patients with sepsis-related blood pressure drop who are going to survive from the ones who are going to die. The area under the ROC curve (AUC) was used to assess the results [23]. The closer the AUC value was to 1, the better the discrimination ability of the predictive model. An AUC > 0.8 indicated that the model was good [24, 25].

The correction of a predictive model was defined as the agreement between the predicted probability and the actual observed probability. In this study, we use calibration chart to evaluate the goodness of fit, with a *P* value greater than 0.05 suggesting an adequate goodness of fit [26].

The clinical effectiveness of the prediction model was evaluated using the DCA curve. Its significance was found in a graph in which the ordinate was the benefit of the evaluation model (All curve) and the abscissa was the risk value of the disease (None curve). Accordingly, the model curve was the curve represented by the model, and the All curve and None curve (the two extreme curves) were used as the criteria. The more the distance of the model curve from the two extreme curves, the better the clinical significance and net benefit conferred to the clinic [25, 27].

The above evaluation was performed using the R software.

### Statistical analysis

The measurement data in this study were not normally distributed tested by twogrps function in R software (except for albumin). The chi-square test was used to evaluate the categorical variables, and the Wilcoxon rank sum test (sample size less than 5000) was used to evaluate the continuous variables with abnormal distribution. For the continuous variable such as albumin, t-test was applied.

## Results

### Analysis and involvement of risk factors of death in patients

A total of 1023 patients were included in this study, divided into 717 in the model group (137 died) and 306 in the validation group (75 died). The global death rate in this study was 20.7% (212/ 1023). There was no significance in baseline characteristics between model group and validation (Table 1).

**Table 1:** Baseline characteristics of the development group and validation group^a^.

Variables	Total (n = 1023) Number (%)	validation (n = 306) Number (%)	Model (n = 717) Number (%)	p
Gender				0.834
no	585 (57)	177 (58)	408 (57)	
yes	438 (43)	129 (42)	309 (43)	
Tumour				0.193
no	829 (81)	240 (78)	589 (82)	
yes	194 (19)	66 (22)	128 (18)	
Peritonitis				0.825
no peritonitis	871 (85)	264 (86)	607 (85)	
peritonitis after surgery	136 (13)	38 (12)	98 (14)	
peritonitis no surgery	16 (2)	4 (1)	12 (2)	
Heart failure ^b^				0.839
no	828 (81)	246 (80)	582 (81)	
yes	195 (19)	60 (20)	135 (19)	
Respiratory failure				0.606
no	848 (83)	257 (84)	591 (82)	
yes	175 (17)	49 (16)	126 (18)	
Consciousness.disturbance				0.253
no	801 (78)	247 (81)	554 (77)	
yes	222 (22)	59 (19)	163 (23)	
Age (years)	70 (57, 80)	70 (58, 80)	70 (57, 80)	0.512
Creatinine (mmol/L)	85 (60, 139.5)	87 (60, 142.25)	84 (60, 139)	0.775
Platelet (10*9/L)	132 (84, 197.5)	129.5 (85, 193.75)	133 (83, 199)	0.968
C-reactive protein (mg/L)	106.5 (58.81, 174.88)	114.97 (60.07, 177.63)	100 (58.1, 172.9)	0.157
White blood cells (10*9/L)	10.8 (6.5, 16.65)	11.1 (6.62, 17.23)	10.7 (6.4, 16.3)	0.289
Bilirubin (μmol/L)	11.3 (7.6, 18.85)	11.35 (7.9, 20.3)	11.1 (7.4, 18.1)	0.197
Albumin (g/L)	28.98 ± 4.94	28.8 ± 4.83	29.06 ± 4.99	0.43

^a^ Continuous variables with abnormal distribution are described by means and quarterbacks and variables with normal distribution are displayed as means ± standard deviation. Categories variables are analyzed by χ2 test and continuous variables with abnormal distribution are analyzed by Wilcoxon rank sum test. For albumin, t-test was applied.

^b^ H-failure, cardiac function grade III or above.

As regard the feasibility of the logistic regression analysis. The P of continuous variable were 0.227, 0.400, 0.593, there was linearity between the continuous variable and logit (p) (S1 Table). Moreover, the variance inflation factors (VIFs) were used to interpret the multicollinearity of all variables (S2 Table). The variance inflation factors (VIFs) were 1.183, 1.083, 1.111, 1.127, 1.126, 1.094, 1.046, 1.050,1.018 respectively, suggesting that there was no multiple-collinearity among the risk factors. All the involved variables could be used in further analysis.

Univariate analysis showed that presence of tumor history, peritonitis, heart failure, consciousness disturbance and respiratory failure had impact on whether survive within 30 days in patients with sepsis-induced drop in blood pressure (Table 2). Also, molecular expression of albumin, bilirubin, C-reaction protein and creatinine and the number of platelets in blood were significantly different between survivors and no survivors.

**Table 2:** Univariate analysis between survivors and no survivors^a^.

Variables	Total (n = 717) Number (%)	Survivors (n = 580) Number (%)	No survivors (n = 137) Number (%)	p
Gender				0.21
no	408 (57)	323 (56)	85 (62)	
yes	309 (43)	257 (44)	52 (38)	
Tumour				< 0.001
no	589 (82)	496 (86)	93 (68)	
yes	128 (18)	84 (14)	44 (32)	
Peritonitis				< 0.001
no peritonitis	607 (85)	502 (87)	105 (77)	
peritonitis after surgery	98 (14)	75 (13)	23 (17)	
peritonitis no surgery	12 (2)	3 (1)	9 (7)	
Heart failure^b^				< 0.001
no	582 (81)	496 (86)	86 (63)	
yes	135 (19)	84 (14)	51 (37)	
Respiratory failure				< 0.001
no	591 (82)	508 (88)	83 (61)	
yes	126 (18)	72 (12)	54 (39)	
Consciousness.disturbance,				< 0.001
no	554 (77)	478 (82)	76 (55)	
yes	163 (23)	102 (18)	61 (45)	
Age	70 (57, 80)	69.5 (55, 79)	75 (64, 83)	< 0.001
Creatinine	84 (60, 139)	80 (59, 125)	120 (68, 204)	< 0.001
Platelet	133 (83, 199)	138 (88, 201.25)	107 (62, 181)	0.002
C-reactive protein	100 (58.1, 172.9)	95.75 (57, 168.94)	119.7 (62.81, 200)	0.001
White blood cells	10.7 (6.4, 16.3)	10.4 (6.57, 16.07)	11.7 (5.8, 17.1)	0.722
Bilirubin	11.1 (7.4, 18.1)	10.85 (7.38, 16.55)	12.3 (7.8, 26.1)	0.022
Albumin	29.06 ± 4.99	29.56 ± 4.79	26.94 ± 5.29	< 0.001

^a^ Continuous variables with abnormal distribution are described by means and quarterbacks and variables with normal distribution are displayed as means ± standard deviation. Categories variables are analyzed by χ2 test and continuous variables with abnormal distribution are analyzed by Wilcoxon rank sum test. For albumin, t-test was applied.

^b^ cardiac function class-III or above.

Then the significant variables in univariate analysis were tested in multivariate logistic regression analysis (Table 3), suggesting that albumin level, creatinine level, presence of cardiac insufficiency, respiratory failure, malignant tumor history, peritonitis requiring surgery and disturbance of consciousness were risk factors for death in patients with sepsis induced drop in blood pressure (Table 3) (P<0.05). Age, which was significant in univariable analysis, was not risk factor in multivariable logistic regression analysis. Thus, seven indexes would be included in the establishment of model after stepwise regression analysis (S3 Table).

**Table 3:** Multivariate logistic regression analysis of involved variables.

	Coefficient (β)	SE	OR (95%CI)	WaldX2	p
(Intercept)	-1.311	0.897	0.270(0.046–1.550)	2.137	0.144
Albumin	-0.069	0.023	0.933(0.891–0.976)	8.886	0.003
Creatine	0.002	0.001	1.002(1.000–1.004)	5.331	0.021
Age	0.003	0.008	1.003(0.988–1.018)	0.106	0.745
Tumor	1.367	0.260	3.924(2.360–6.546)	27.720	<0.001
Peritonitis after surgery	0.335	0.314	1.398(0.743–2.555)	1.138	0.286
Peritonitis no surgery	3.031	0.754	20.719(5.141–106.657)	16.184	<0.001
Heart failure	0.973	0.251	2.646(1.614–4.324)	15.054	<0.001
Respiratory failure	1.418	0.265	4.130(2.460–6.976)	28.569	<0.001
Consciousness disturbance	0.993	0.233	2.701(1.709–4.264)	18.207	<0.001

### Nomogram establishment for predicting risk of death

**Fig 1:**
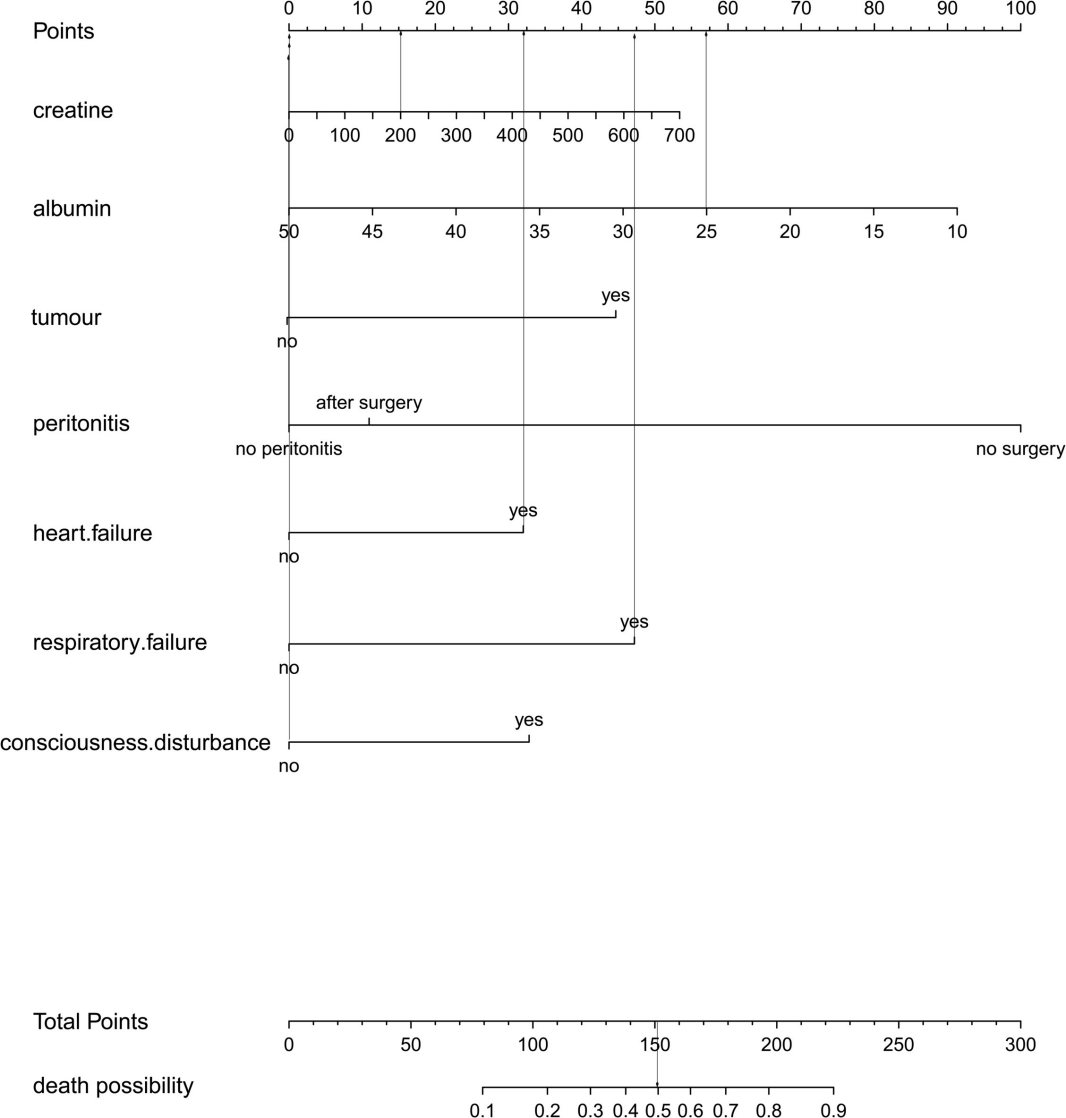
Risk-prediction nomogram for death in patients with sepsis-induced blood pressure drop. Arrows means matching results between the variables (from a patient as an example) and the point reference. The patients had no tumor history, no peritonitis, no consciousness disturbance, but had respiratory failure and heart failure. The levels of creatinine and albumin were 200 mmol/L and 25 g/L respectively.

According to univariate analysis and multivariate logistic regression analysis and stepwise regression analysis, seven independent risk factors were included in the prediction model to develop an individualized nomogram prediction model (Fig 1), which should be interpreted as follows. A patient, for example, with sepsis-related blood pressure drop, cardiac function class III or above at the time of the onset (32 points), respiratory failure (47 points), without peritonitis requiring surgery (0 point), a creatinine level of 200 mmol/L (15.5 points), without tumor history (0 point), without consciousness disturbance (0 point), albumin level of 25g/L (57 points), had a total score of 151.5 points compared to the nomogram, corresponding to a 50% risk of death, thus having a high risk of death.

### Evaluation of predicting death in model group

**Fig 2:**
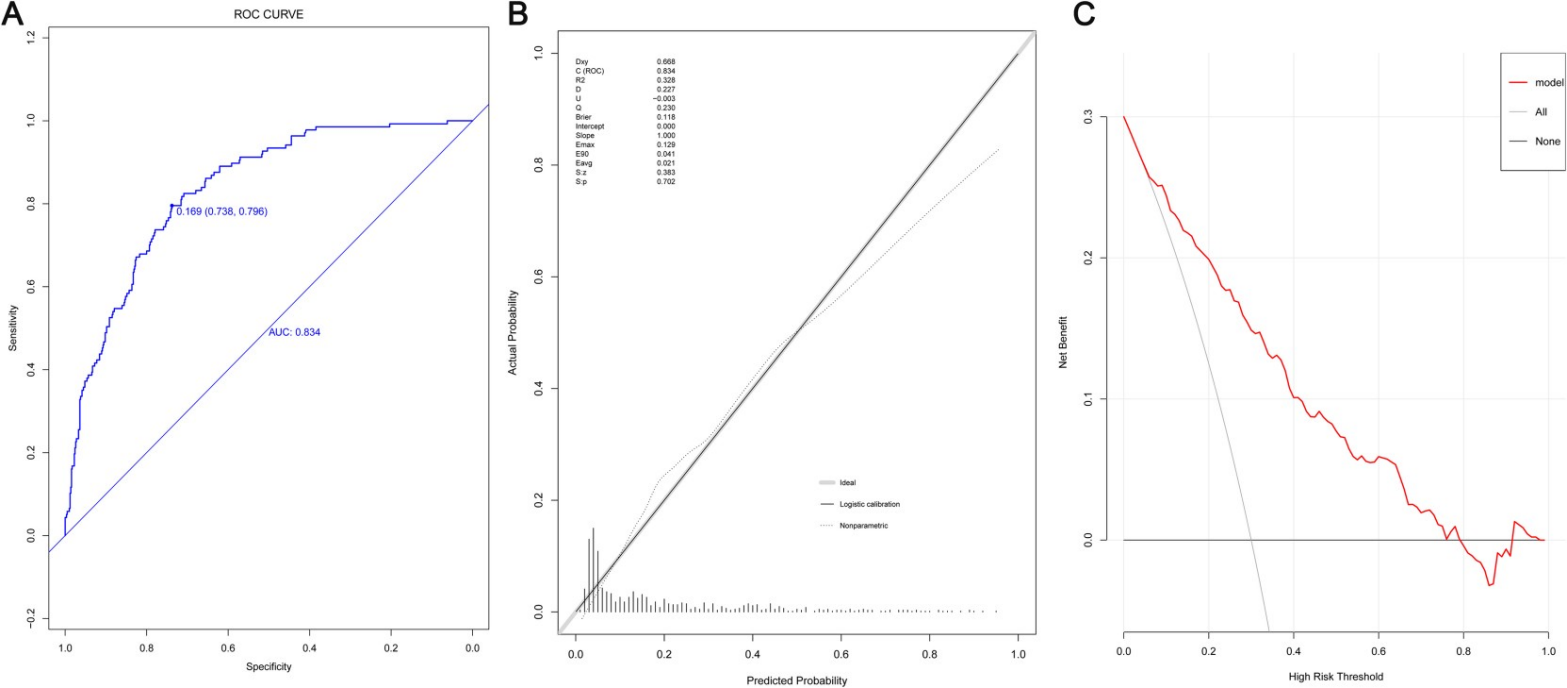
Evaluation of prediction model in model group. (A) ROC curves; (B) Calibration chart; (C) DCA curves.

Evaluation of the model discrimination: The area under the ROC curve of the model was 0.834 (Fig 2A), suggesting a good discrimination. At the optimal cut off point of 0.169, the model owned high specificity (73.8%) and sensitivity (79.6%). The correction plot (Fig 2B) illustrated that the fitting logistic curve (solid line) of the model group highly overlapped the standard curve, with a *P* value of 0.702, suggesting an adequate goodness of fit. The DCA curve (Fig 2C) was used for this evaluation. The curve of the model group was above the two extreme curves, without any intersection with the two extreme curves in the risk interval of 10% -80%, suggesting that the model this study possessed clinical effectiveness.

### Validation of prediction model in validation group

**Fig 3:**
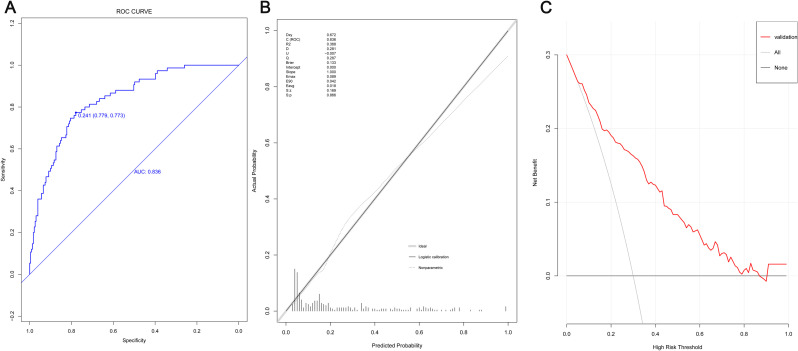
Validation of prediction model in validation group. (A) ROC curve; (B) Calibration chart; (C) The DCA curve.

The model validation was also evaluated through the ability of discrimination (Fig 3A), correction (Fig 3B), and clinical significance (Fig 3C). In these figures, the ROC curve, correction diagram and DCA curve of the validation group were plotted. The area under the ROC curve was 0.836, suggesting a good discrimination. The P value of the correction diagram of the validation group was 0.866, suggesting that the model fitted the validation group. The DCA curve of the validation group had a good clinical value within the risk interval of 10% -90%.

## Discussion

This study showed that the independent risk factors for death in patients with sepsis-related blood pressure drop were the presence of cardiac insufficiency class III or higher at the time of the onset, presence of respiratory failure, consciousness disturbance, malignant tumor history, peritonitis, albumin level, and creatinine level.

The real experience in clinical practice suggests that the blood pressure drops due to sepsis is associated with adverse clinical outcomes [28–31]. This pathology is more life-threatening than the sepsis alone, thus, a study evaluating this population would be of great significance. In this work, an individualized nomogram to predict the death within 30 days among patients with sepsis-related blood pressure drop was established. The ROC curve, correction curve and DCA curve were used to evaluate the discrimination, prediction accuracy and clinical effectiveness of the model, which were all significant. Furthermore, a good significance was demonstrated in the validation group, suggesting that this model might be beneficial in clinical practice.

Sepsis, caused by infection, usually results in disorders of immune response and clinical symptoms displaying dysfunction in kidney, heart and coagulation, consequently increasing the risk rate of death. In addition, the patients with sepsis induced drop in blood pressure show symptoms of hypoperfusion. However, the patients involved in this study include patients with sepsis shock and patients only with sepsis-induced drop in blood pression (could not meet the criteria of sepsis because of persistence duration of low blood pressure and lactic acid level) [15]. Theoretically, some patients in this study are milder than patients with sepsis shock.

Therefore, factors involved in sepsis shock mentioned above may be risk factors of death in patients with sepsis induced drop in blood pressure. As showed in our results, heart dysfunction is the death related risk factor, which is same to previous work [32]. In patients with sepsis-induced hypotension, the treatment requires fluid resuscitation, whereas in patients with cardiac insufficiency, fluid sensitivity increases, and improper fluid resuscitation leads to increased mortality. Peritonitis before admission to wards in this study, signs requiring surgery (Surgery for peritonitis), such as those generally caused by perforation, strangulated intestinal obstruction, necrotizing appendix and other surgical consequences, often leading to sepsis with blood pressure drop and requiring surgical treatment., Surgery is an appropriate treatment in patients with peritonitis, but it is evident that the injury from the disease itself, combined with the injury from surgery, lead to increased mortality [33, 34]. If no surgery is performed, the primary disease is not resolved, resulting in a dramatic deterioration of the patient condition. Furthermore, the survival rate of patients that do not undergo surgery is very low, based on the P values and ORs. In summary, the death risk of patients with sepsis induced hypotension increases in patients with peritonitis, especially in those patients without surgery involvement. “Tumor factors” is referred to patients with previous or current malignant tumors, regardless of the previous or current use of chemotherapy, surgery and other therapies. In patients, the effect of tumor itself and that of chemotherapy is already understood. Both tumors and chemotherapy affect the patient immunity. Existing studies demonstrated that tumors are one of the factors influencing the prognosis of patients with sepsis/septic shock [11]. As regard the role of albumin, some studies reported that albumin is an important factor affecting the critical prognosis of patients with sepsis/septic shock [35–38]. It is reasonable that such patients with respiratory failure will suffer from hypoxia and increased in mortality. Even if they were treated with ventilator, complications in the treatment will also led to increased mortality. The weakening of consciousness which means the decrease of self-protection function, may results in some complications, such as asphyxia and leads to the increase of mortality. In clinic, creatinine was a common index that was used to judge renal function, while patients with renal dysfunction had increased mortality.

Comparing to previous studies, not only the risk factors for short-term mortality in patients with sepsis-induced hypotension were found in this study, but these factors were further analyzed to derive specific scores for each of the influencing factors and present them on nomogram [17, 18]. The total score was calculated according to the specific scores of each influencing factor, and the death probability was deduced. The death risk was no longer an abstract fact, but more specific, which is an advantage not present in the other current clinical critical evaluation tools.

This study possesses some limitations: (1) it is a retrospective study, thus, a congenital deviation in the data collection is present; (2) this study is a single-centered study, thus lacking data verification with other centers; (3) some indicators were not included in this study due to data loss, such as lactate and procalcitonin level, of which more than 30% of the data was missing, leaving no room for interpolation; (4) although most patients with biliary obstruction, ureteral obstruction or those requiring drainage were subjected to a drainage procedures, a small number of patients refused drainage and their specific analyses were not performed. Hence, patients who required drainage but they were not subjected to it, might have a higher risk according to this model. Our plan is to continue to collect the data of patients with blood pressure drop caused by sepsis in subsequent studies in the next decade. Additionally, our future aim is to reduce data loss, thereby improving the collection of laboratory indicators in such patients and try to acquire additional data. Finally, it is of utmost importance to invite other centers to participate in our study.

## Conclusion

This study developed a prediction model to evaluate the risk of short-term mortality in patients who suffer from sepsis-associated blood pressure drop. Through this model, the short-term mortality risk of these patients can be accurately evaluated, providing a more concrete and specific reference for clinicians.

## Supporting information

S1 Table The linear relationship analysis between continuous variables and logit (p). (DOCX)

S2 Table Variance inflation factor of variables. (DOCX)

S3 Table Stepwise regression analysis of involved variables. (DOCX)

S1 Checklist *PLOS ONE* clinical studies checklist. (DOCX)

S1 Code (DOCX)

S1 Data (CSV)

S2 Data (CSV)

S1 File (DOCX)
