# Supplementary material for: Establishment and validation of a predictive model for mortality within 30 days in patients with sepsis-induced blood pressure drop: A retrospective analysis
Source: PLoS One. 2021 May 20;16(5):e0252009. doi: 10.1371/journal.pone.0252009 (PMC8136670; doi:10.1371/journal.pone.0252009)
Supplement: S1 Table — (DOCX) [file pone.0252009.s001.docx]

| Variables | Pr (>\|z\|) |
| --- | --- |
| Albumin | 0.227 |
| Creatinine | 0.400 |
| Age | 0.593 |

S1 Table. The linear relationship analysis between continuous variables and logit (p).
