# Supplementary material for: Establishment and validation of a predictive model for mortality within 30 days in patients with sepsis-induced blood pressure drop: A retrospective analysis
Source: PLoS One. 2021 May 20;16(5):e0252009. doi: 10.1371/journal.pone.0252009 (PMC8136670; doi:10.1371/journal.pone.0252009)
Supplement: S2 Table — (DOCX) [file pone.0252009.s002.docx]

S2 Table. Variance inflation factor of variables

| Variables | Variance inflation factor |
| --- | --- |
| Respiratory failure | 1.183 |
| Creatinine | 1.083 |
| Albumin | 1.111 |
| Heart failure | 1.127 |
| Age | 1.126 |
| Consciousness.disturbance | 1.094 |
| Tumor | 1.046 |
| Peritonitis after surgery  Peritonitis no surgery | 1.050  1.018 |
