# Supplementary material for: Establishment and validation of a predictive model for mortality within 30 days in patients with sepsis-induced blood pressure drop: A retrospective analysis
Source: PLoS One. 2021 May 20;16(5):e0252009. doi: 10.1371/journal.pone.0252009 (PMC8136670; doi:10.1371/journal.pone.0252009)
Supplement: S3 Table — (DOCX) [file pone.0252009.s003.docx]

S3 Table. Stepwise regression analysis of involved variables.

|  | Coefficient(B) | SE | OR(95%CI) | WaldX2 | p |
| --- | --- | --- | --- | --- | --- |
| (Intercept) | -1.135 | 0.717 | 0.321(0.078-1.309) | 2.506 | 0.113 |
| Albumin | -0.069 | 0.023 | 0.933(0.890-0.976) | 9.012 | 0.003 |
| Creatine | 0.002 | 0.001 | 1.002(1.000-1.004) | 5.345 | 0.020 |
| Tumor | 1.371 | 0.259 | 3.943(2.372-6.574) | 27.963 | <0.001 |
| Peritonitis after surgery | 0.334 | 0.314 | 1.397(0.743-2.553) | 1.136 | 0.286 |
| Peritonitis no surgery | 3.054 | 0.748 | 21.202(5.310-108.350) | 16.638 | <0.001 |
| Heart failure | 0.977 | 0.251 | 2.659(1.623-4.340) | 15.233 | <0.001 |
| Respiratory failure | 1.442 | 0.256 | 4.227(2.563-7.002) | 31.742 | <0.001 |
| Consciousness disturbance | 1.002 | 0.231 | 2.724(1.729-4.288) | 18.766 | <0.001 |
