# Supplementary material for: Establishment and validation of a predictive model for mortality within 30 days in patients with sepsis-induced blood pressure drop: A retrospective analysis
Source: PLoS One. 2021 May 20;16(5):e0252009. doi: 10.1371/journal.pone.0252009 (PMC8136670; doi:10.1371/journal.pone.0252009)
Supplement: S1 Code — (DOCX) [file pone.0252009.s005.docx]

#load R package

library(foreign)

library(pROC)

library(caret)

library(CBCgrps)

library(tidyverse)

library(car)

library(rms)

#set up path

setwd("D:/R work")

#read data

dev<-read.csv("dev.csv",header=TRUE,sep=",",na="")

vad<-read.csv("vad.csv",header=TRUE,sep=",",na="")

#remove missing values

dev<-na.omit(dev)

vad<-na.omit(vad)

#content given

dev$gender<-factor(dev$gender,labels=c("no","yes"))

dev$tumour<-factor(dev$tumour,labels = c("no","yes"))

dev$peritonitis<-factor(dev$peritonitis,labels = c("no peritonitis","after surgery","no surgery"))

dev$H.Failure<-factor(dev$H.Failure,labels = c("no","yes"))

dev$R.Failure<-factor(dev$R.Failure,labels = c("no","yes"))

dev$consciousness.disturbance<-factor(dev$consciousness.disturbance,labels = c("no","yes"))

vad$gender<-factor(vad$gender,labels=c("no","yes"))

vad$tumour<-factor(vad$tumour,labels = c("no","yes"))

vad$peritonitis<-factor(vad$peritonitis,labels = c("no peritonitis","after surgery","no surgery"))

vad$H.Failure<-factor(vad$H.Failure,labels = c("no","yes"))

vad$R.Failure<-factor(vad$R.Failure,labels = c("no","yes"))

vad$consciousness.disturbance<-factor(vad$consciousness.disturbance,labels = c("no","yes"))

#baseline characteristics of the development group and validation group

vad$group<-1

dev$group<-2

data<-rbind(dev,vad)

table1<-twogrps(data,gvar="group",c("gender","tumour","peritonitis","H.Failure","R.Failure",

"consciousness.disturbance","age","cr","plt","crp",

"wbc","bilirubin","albumin"))

print(table1,quote = T)

#Univariate analysis between survivors and no survivors

table2<-twogrps(dev,gvar = "death",c("gender","tumour","peritonitis","H.Failure","R.Failure",

"consciousness.disturbance","age","cr","plt","crp",

"wbc","bilirubin","albumin"))

print(table2,quote = T)

#linearity verification

fit<-glm(death~albumin+cr+age+tumour+peritonitis+H.Failure+R.Failure+consciousness.disturbance, data=dev, family=binomial(link="logit"))

prob<-predict(fit,type = "response")

logitp<-log(prob/(1-prob))

boxTidwell(logitp~albumin+cr+age,~tumour+peritonitis+H.Failure+R.Failure+consciousness.disturbance,data = dev)

vif.dia<-vif(lm(death~age+cr+albumin+tumour+peritonitis+H.Failure+R.Failure+consciousness.disturbance,data = dev))

summary(vif.dia)

sort(vif.dia,decreasing = TRUE)

#multivariate logistic regression analysis(The significant variables in univariate)

modelA<-glm(death~age+cr+albumin+tumour+peritonitis+H.Failure+R.Failure+consciousness.disturbance,

data=dev,family=binomial(link="logit"))

summary(modelA)

#display R value

cbind(coef= coef(modelA),confint(modelA))

exp(cbind(OR= coef(modelA),confint(modelA)))

#forward stepwise method

modelAIC <- step(object=modelA,direction="backward")

summary(modelAIC)

cbind(coef= coef(modelAIC),confint(modelAIC))

exp(cbind(OR= coef(modelAIC),confint(modelAIC)))

#model according to the selected variables

modelA2<-glm(death~cr+albumin+tumour+peritonitis+H.Failure+R.Failure+consciousness.disturbance,

data=dev,family=binomial(link="logit"))

dev$predmodelA<- predict(newdata=dev,modelA2,"response")

#draw nomogram

dev$heart.failure<-dev$H.Failure

dev$respiratory.failure<-dev$R.Failure

ddist <- datadist(dev)

options(datadist='ddist')

func1 = function(x) 1/(1+exp(-x))

modelA3<-lrm(death~cr+albumin+tumour+peritonitis+H.Failure+R.Failure+consciousness.disturbance,data=dev)

nomomodelA3 <- nomogram(modelA3,fun= func1 ,fun.at = seq(0.1,1,by=0.1),funlabel= "Diagnostic possibility")

plot(nomomodelA3)

#draw ROC of dev

gmodelA <- roc(death ~predmodelA, data = dev)

plot(gmodelA, print.auc=TRUE, print.thres=TRUE,main = "ROC CURVE", col= "blue",print.thres.col="blue",identity.col="blue",

identity.lty=1,identity.lwd=1)

#draw calibration chart of dev

val.prob(dev$predmodelA,dev$death)

#draw DCA of dev

library(rmda)

dcamodelA<- decision_curve(death ~cr+albumin+tumour+peritonitis+H.Failure+R.Failure+consciousness.disturbance, data= dev, family = binomial(link ='logit'),thresholds= seq(0,1, by = 0.01),confidence.intervals = 0.95,study.design = 'case-control', population.prevalence = 0.3)

List<- list(dcamodelA)

plot_decision_curve ( List,curve.names=c('model'),cost.benefit.axis =FALSE, col= c('red'), confidence.intervals = FALSE,standardize = FALSE)

#draw ROC of vad

modelB2<-glm(death~cr+albumin+tumour+peritonitis+H.Failure+R.Failure+consciousness.disturbance,

data=vad,family=binomial(link="logit"))

vad$predmodelB<-predict(newdata=vad,modelB2,"response")

gmodelB<- roc(death ~predmodelB, data = vad)

plot(gmodelB, print.auc=TRUE, print.thres=TRUE,main = "ROC CURVE", col= "blue",print.thres.col="blue",identity.col="blue",

identity.lty=1,identity.lwd=1)

#draw calibration chart of vad

val.prob(vad$predmodelB,vad$death)

#draw DCA of vad

dcamodelB<- decision_curve(death ~cr+albumin+tumour+peritonitis+H.Failure+R.Failure+consciousness.disturbance, data= vad, family = binomial(link ='logit'),thresholds= seq(0,1, by = 0.01),confidence.intervals = 0.95,study.design = 'case-control', population.prevalence = 0.3)

List<- list(dcamodelB)

plot_decision_curve ( List,curve.names=c('validation'),cost.benefit.axis =FALSE, col= c('red'), confidence.intervals = FALSE,standardize = FALSE)

**Please note that：**

**H** **Failure means Heart failure**

**R Failure means Respiratory failure**
