# Supplementary material for: Establishment and validation of a predictive model for mortality within 30 days in patients with sepsis-induced blood pressure drop: A retrospective analysis
Source: PLoS One. 2021 May 20;16(5):e0252009. doi: 10.1371/journal.pone.0252009 (PMC8136670; doi:10.1371/journal.pone.0252009)
Supplement: S1 File — (DOCX) [file pone.0252009.s008.docx]

Dev：Modeling data

Vad：Validation data

Variable interpretation：

gender:0,male;1,female

peritonitis:0,no;1,yes after surgury;2,yes no surgery

R Failure:respiratory failure,0,no;1,yes

H failure:heart failure,0,no;1,yes

Disturbance of consciousness:0,no;1,yes

Tumour:0,no;1,yes
